# The Possible Contribution of being Born by Cesarean Section to Developing Childhood Overweight and Obesity in Later Life

**DOI:** 10.1055/s-0041-1731381

**Published:** 2021-07-27

**Authors:** Anibal Faúndes, Laura Miranda, Silvana Ferreira Bento

**Affiliations:** 1 Department of Obstetrics and Gynecology, Universidade Estadual de Campinas, Campinas, São Paulo, Brazil; 2 Campinas Centre for Research in Human Reproduction (Cemicamp), Campinas, São Paulo, Brazil

## Introduction

Obesity is now a major global epidemic. In 2016, 39% of adults worldwide ≥ 18 years old were overweight, and 13% were obese, as reported by the World Health Organization (WHO). 1 This current scenario is compounded in high-income countries such as the United States, where 31% of the population has a body mass index (BMI) > 30 kg/m ^2^ and 50% have a waist circumference ≥ 88 cm, in the case of women, or ≥ 102 cm in the case of men. 2 Overweight and obesity are rapidly becoming a major health issue, as these conditions are associated with severe chronic diseases such as diabetes, hypertension, and cardiovascular diseases in general. 3

While it is largely agreed that obesity and overweight are the consequences of an unhealthy diet, mainly due to an increasing use of processed and preserved foods together with minimal physical activity, an association between cesarean delivery and obesity in later life has also been suggested. Cesarean section rates continue to increase well beyond what could be medically justified, and several studies conducted in different countries have found an association between being born by cesarean section and developing obesity in childhood or adulthood. 4

## Cesarean Section and the Development of Overweight and Obesity

The Growing Up Today Study (GUTS), a large prospective study of individuals followed-up from childhood through early adulthood in the United States, evaluated obesity as defined by the cutoff limit of BMI ≥ 30 kg/m ^2^ , in accordance with the WHO criteria. The multivariate adjusted risk ratio (RR) for obesity was 1.15 for infants delivered by cesarean section compared with those born by vaginal delivery (95% confidence interval [CI]: 1.06–1.26; *p* = 0.002). The multivariate adjusted RR for obesity according to age group was: 1.23 (95%CI: 1.03–1.46) at ages between 9 and 12 years old, 1.16 (95%CI: 1.03–1.31) at ages between 13 and 18 years old, and 1.10 (95%CI: 0.98–1.24) at ages between 19 and 24 years old ( *p-value* for heterogeneity = 0.13). The associations were similar both for females (1.12; 95%CI: 0.99–1.27) and for males (1.18; 95%CI: 1.04–1.34) ( *p-value* for heterogeneity = 0.62). 5

Three birth cohorts in the Brazilian city of Pelotas, state of Rio Grande do Sul, were followed-up into adulthood using a very similar methodology. All babies born in 1982, 1993 and 2004 whose mothers lived in the city were recruited to the studies. Fat mass index and BMI z-scores in the offspring were strongly and positively associated with cesarean birth in the crude analysis. However, following adjustment for socioeconomic characteristics and maternal factors such as BMI, parity, age, and smoking during pregnancy, the association disappeared, except for the group of 30-year-old women. In these women from the 1982 cohort, cesarean section remained associated with fat mass index (β = 0.82; 95%CI: 0.32–1.32) and BMI z-score (β = 0.15; 95%CI: 0.03–0.28). Following adjustment for potential confounders, the association persisted, indicating higher fat mass index and BMI at the age of 30 years old in those born by cesarean section compared with those delivered vaginally. 6

The Project Viva enrolled participants between April 1999 and July 2002 in eastern Massachusetts, USA, forming a longitudinal prebirth cohort of mother-offspring pairs. In the children delivered by cesarean section, there was a 2-fold likelihood of developing obesity, with a higher BMI (∼ 0.2 z-score units higher) and higher sum of triceps plus subscapular skinfold thicknesses (∼ 1 mm) at the age of 3 years old compared with children delivered vaginally. These differences between the two modes of delivery persisted following adjustment for key potential confounding factors including maternal BMI and birthweight. 7

In South Africa, an analysis of young adults aged between 21 and 24 years old from a longitudinal urban birth cohort showed that having been born by cesarean section was associated with obesity in early adulthood. In the crude analysis, birth by cesarean section was associated with an almost 2-fold increase in the risk of obesity among young people between 21 and 24 years old. Following adjustment for gender, birthweight, and the mother's parity and education level at delivery as possible confounders, the strength of the association was maintained. 8

No such association was found, however, in a large, contemporary, prospective, longitudinal cohort study, the Millennium Cohort Study (MCS), conducted in the United Kingdom, which found no statistically significant difference in BMI at the ages of 3 and 14 years old in infants born by planned cesarean section compared with those born by vaginal delivery. Furthermore, there was no difference in body fat percentage at 7 and 14 years old between infants born by planned cesarean section and those born by vaginal delivery. 9

A very large systematic review and meta-analysis conducted to investigate associations between mode of delivery and adult BMI, overweight, and obesity found that the mean BMI was 0.44 kg.m ^−2^ greater in subjects delivered by cesarean section compared with those delivered vaginally. The increased odds of overweight and obesity > 20% applied to both genders. 10

## The Role of the Gut Microbiome as a Mechanism of Action in Overweight and Obesity

As accumulated global experience appears to confirm the association between being born by cesarean section and overweight and obesity in later life, a possible explanation for this association may lie in the limited microbial diversity reported in offspring delivered by cesarean section compared with that of those born by vaginal delivery. 11 12 Infants born by vaginal delivery are exposed to microorganisms mainly in the birth canal or in the vaginal environment, while those delivered by cesarean section are exposed to microflora on the mother's skin. 13 This is presumed to persist into adulthood. 5

Experiments in rodents have shown the important role of microorganisms in the mechanism of obesity. Transferring fecal microbial content from normally raised mice to adult germ-free mice leads to a very rapid and voluminous increase in body fat within between 10 and 14 days, even when food consumption is reduced. 14 There are several mechanisms involved in these changes, including microbial fermentation of dietary polysaccharides that cannot be digested by mice, the contribution of monosaccharide and fatty acids to intestinal absorption, their metabolism in the liver, and the regulation of host genes that promote the deposition of lipids in adiposity. 14

Infants delivered by cesarean section, particularly elective cesarean section, are generally not exposed to their mother's vaginal and fecal microbiota, which helps shape the initial composition of an infant's gut microbiota. 13 The diverseness and richness of their gut microbiome has been found to be poor. 15 According to some studies, the gut microbiota of infants born by cesarean section may have a tendency to harvest more dietary nutrients, thus predisposing them to being overweight or obese. 16 17

## Conclusion

Although the effect of the rise in the number of cesarean sections on the increasing rates of overweight and obesity may be minimal, it would nonetheless appear to constitute one more factor contributing to obesity, a worldwide health concern.
